# Importance of the Difference in Surface Pressures of the Cell Membrane in Doxorubicin Resistant Cells That do not Express Pgp and ABCG2

**DOI:** 10.1007/s12013-012-9497-0

**Published:** 2013-01-13

**Authors:** Charlotte Bell, Claire Hill, Christopher Burton, Adam Blanchard, Freya Shephard, Cyril Rauch

**Affiliations:** 1 University of Nottingham, School of Veterinary Medicine and Science, Sutton Bonington Campus, Sutton Bonington, Leicestershire, LE12 5RD UK; 2 University of Birmingham, School of Medicine and Dentistry, Edgbaston, Birmingham, B15 2TT UK

**Keywords:** Drug resistance, Pharmacokinetic, Membrane, Drug transporter, P-glycoprotein, MDR

## Abstract

P-glycoprotein (Pgp) represents the archetypal mechanism of drug resistance. But Pgp alone cannot expel drugs. A small but growing body of works has demonstrated that the membrane biophysical properties are central to Pgp-mediated drug resistance. For example, a change in the membrane surface pressure is expected to support drug–Pgp interaction. An interesting aspect from these models is that under specific conditions, the membrane is predicted to take over Pgp concerning the mechanism of drug resistance especially when the surface pressure is high enough, at which point drugs remain physically blocked at the membrane level. However it remains to be determined experimentally whether the membrane itself could, on its own, affect drug entry into cells that have been selected by a low concentration of drug and that do not express transporters. We demonstrate here that in the case of the drug doxorubicin, alteration of the surface pressure of membrane leaflets drive drug resistance.

## Introduction

In 2007, the American Cancer Society report concluded that cancer kills ~7 m people a year worldwide (1 in 8 deaths). One of the major concerns in this field is that many cancers fail to respond to chemotherapy, by acquiring multi-drug resistance (MDR), to which has been attributed the failure of treatment in over 90 % of patients with metastatic cancer [1]. Furthermore, it is now recognized that cancer aggressiveness, i.e. the metastatic potential of tumours, is related to their resistance to chemotherapy [2, 3].

One major form of resistance to chemotherapy has been correlated with the presence of membrane molecular “pumps” that actively transport drugs out of the cell. Historically, it was in 1973 that Dano Keld suggested that the mechanism of resistance was due to an outward efflux of drugs that “vacuum clean” drugs from cells [4]. This hypothesis gained credence 3 years later when P-glycoprotein (Pgp) was identified as a membrane protein over-expressed in MDR cancer cells that actively extrude membrane-embedded drugs [5]. Since then and further to an important body of works the molecular basis of Pgp is now defined with remarkable precision [6]. Although the molecular model of Pgp has permitted a representation of MDR in agreement with the usual concepts issued from the field of biochemistry, how a single protein can expel structurally different drugs is still poorly understood [7]. Accordingly, models of drug resistance were suggested to complete the Pgp theory, assuming a fundamental role for the cell membrane biophysical properties to sustain drug pumping in resistant cells [8–11]. It is noteworthy that under certain conditions the asymptotic forms of these membrane-based theories can also explain drug resistance without a pumping mechanism but solely by considering the biophysical properties of the membrane and related interaction with drugs.

Suggesting the involvement of the cell membrane in drug delivery, efficacy, or resistance is neither new nor a mystery [12]. This is particularly true concerning the membrane biomechanical properties as not only the basic principles of pharmacokinetic consider as central the role of surface pressure (or similarly surface tension) in the transverse movement of drugs across the membrane but also; the first seminal study on drug resistance performed more than 40 years ago demonstrated the correlation between the molecular weight (*M*_*W*_) of drugs and levels of resistance [13], suggesting similarly a role for the surface pressure [14]. The role of membrane surface pressure was also investigated more recently using the Langmuir–Blodgett technique and lipid monolayer film extracts from sensitive or Pgp-expressing resistant cells [15]. It was found that upon doxorubicin incubation, the surface pressure develops more and is stable in extracts from Pgp-expressing resistant cells, suggesting stronger interaction between doxorubicin and lipids in this case.

Although a number of theoretical and experimental results suggest that the cell membrane and its related mechanical properties are paramount in drug resistance, little (if any) has been done in living cells. The major problem in this case is that transporters are also expressed in drug resistant cells and from living cell studies it is almost impossible to differentiate between pumping or membrane effects. Only reconstituted systems for transporters [16], or lipid extracts as above, are available to study a particular parameter in drug resistance. However, due to their complex nature cells can display major differences with model systems [17].

To resolve this issue and determine whether the membrane biomechanical properties can be fundamentally involved in drug resistance, we have selected drug resistant K562 cell (DRK562) with 10 nM doxorubicin over a period of 6 months. With such a low concentration of doxorubicin no Pgp or ABCG2 were identified albeit resistance to doxorubicin was measured. We demonstrate here that mechanical effects associated with changes in the surface pressures of membrane leaflets are indeed present in DRK562 versus K562.

## Materials and methods

### Cell culture

K562 human erythroleukemia cell lines kindly provided by Pluen [18]. Both K562 and DRK562 were separately cultured in RPMI supplemented by 10 % FBS and 2 mM l-glutamine. Cells were maintained at 37 °C in a 5 % CO_2_ atmosphere. DRK562 were further incubated with a constant 10 nM of Doxorubicin. At confluence, 50 ml of media containing either K562 or DRK562 cells were centrifuged at 1,200 rpm for 10 min to collect and re-suspend the cell pellets into the desired volume of cell culture media and in the required presence of doxorubicin in the case of DRK562.

### FACS

Mouse monoclonal primary antibodies against Pgp (clone UIC2—abcam) or ABCG2 (clone 5D3—abcam) were used on fixed cells with cold PFA4 % (w/v) for 30 min. Membrane permeabilization was carried out using 0.05 % (w/v) saponin for 45 min. 0.2 % BSA (w/v) was subsequently used to block unspecific interactions. BSA was also present at the same concentration when incubating Pgp antibodies conjugated to phycoerythrin (*E*_x_/*E*_m_ = 488/578) or FITC (*E*_x_/*E*_m_ = 488/519) for an hour with cells. Cells were then subsequently washed three times in PBS and individual fluorescence intensity of cells were analysed by cytofluorimetry using a FACS (BD FACS Canto II). 10,000 events were recorded for each sample at a medium flow rate and subsequently analysed (FACSDiva Software).

### Western blotting

Incubations were terminated by washing with ice-cold PBS containing orthovanadate (Na3VO4) at 0.4 mM and whole cell lysates prepared in lysis buffer [63.5 mM Tris_HCl, pH 6.8, 10 % glycerol (vol/vol), 2 % SDS (wt/vol), 1 mM Na3VO4, 1 mM AEBSF, 50_g/ml leupeptin, 5 % mercaptoethanol (vol/vol), and 0.02 % bromophenol blue (wt/vol)]. The protein content of the cell lysate was measured using the Bradford test (Sigma) and a spectrophotometer (BMG FluoStar Optima), and equal quantities of protein (30–60 μg/lane) were resolved using SDS-PAGE (10, 7, or 4–12 %). The gel was then transferred onto Hybond-P membrane (Amersham, Piscataway, NJ) that was then blocked with non-fat dry milk or BSA at 5 % (wt/vol) in PBS-Tween (1:1,000 vol/vol). For immunodetection, the ABCG2 (clone 5D3—abcam) monoclonal primary antibody and the Pgp (ab98322—abcam) rabbit polyclonal antibody were used at a concentration of 1:1,000 (vol/vol) in PBS–Tween for 1 h. The membrane was subsequently washed five times for 10 min each in PBS–Tween. HRP-conjugated antibody was added at a concentration of 1:10,000 (vol/vol) in PBS–Tween for 1 h, and the membrane was washed five times for 10 min each in PBS–Tween before the chemiluminescence reaction was performed using ECL Plus (Amersham). Protein levels were examined using Hyperfilm (Amersham).

### Doxorubicin resistance levels deduced from doxorubicin-induced cell death

Cells were stained using Dead Cell Apoptosis Kit with Annexin V Alexa Fluor^®^ 488 (*E*_x_/*E*_m_ = 495/519 nm) and Propidium Iodide (*E*_x_/*E*_m_ = 536/617 nm) (Invitrogen) following manufacturer instructions. The FACS was immediately used to determine cell viability and/or stage of apoptosis. 10,000 events were recorded for each sample at a medium flow rate and subsequently analysed (FACSDiva Software). Data were compared against a logistic equation: $\hbox{min} + (\hbox{max} - \hbox{min} )/\left[ {1 + \left( {D_{\text{OX}} /EC_{50} } \right)^{H} } \right]$, to determine the effective concentrations. In the last relation, “min” and “max” relate to the minimal and maximal percentages of living cells, “*D*_ox_” is the doxorubicin concentration, “EC_50_” the effective doxorubicin concentration needed to kill 50 % cells and *H* the Hill coefficient.

### Doxorubicin diffusion across the cell membrane

Two-hundred millilitre of K562 and 200 ml of DRK562 were centrifuged at 1,200 rpm for 10 min. Cell pellets (~250 μl for each cell type) were collected and re-suspended in 300 μl PBS. Note that a cell pellet of 250 μl contains about 125 million cells. The cells were washed two times with 300 μl PBS after centrifugation at 7.6 g for 30 s. Cells were then subsequently plated onto a 96-well plate at constant ratio of 10 μl pellet volume/90 μl PBS per well providing about 5 million cells per well. 100 μl PBS–doxorubicin were subsequently injected per well at the desired concentration (no doxorubicin was present in the injected solution for controls). Fluorescence intensities were collected over time using a fluorescence plate reader (BMG FluoStar Optima) using the 488 /620 nm excitation–emission wavelengths. When needed, triton X-100 was injected in wells at the concentration of 0.05 % (v/v). Due to the inherent difficulty to measure the different quantum yields of doxorubicin in cells—that is a prerequisite for an accurate description of how the fluorescence intensity changes along the time—a heuristic model was devised instead. The normalized fluorescence intensities (see Fig. 3a, b) were mono- or bi-phasic and we assumed that they could be represented by the sum two classical “exponential-type rise to the max” functions:

1 $$ \Updelta \tilde{I}(t) = \frac{I(t)}{{I_{0} }} - 1 = a\left( {1 - {\text{e}}^{ - bt} } \right) + c\left( {1 - {\text{e}}^{ - dt} } \right),{\text{ is:}} $$

where, $I_{0}$, is the initial intensity. Matching experimental data against Eq. 1 always provided good correlations ($R^{2} > 0.980$; Fig. 3c). After the initial extinction we found associated with doxorubicin binding to cell membrane, the initial parts of fluorescence recovery, i.e. ascending parts of intensities, were supposed to be related to doxorubicin crossing the membrane. Thus, determining the intensity changes over time in this region provided the kinetics of doxorubicin transmembrane diffusion. We therefore derived the formula for ${\text{d}}\Updelta \tilde{I}/{\text{d}}t$ in the region that contains the flexion point where mathematically: $\left( {{\text{d}}^{2} \Updelta \tilde{I}/{\text{d}}t^{2} } \right)_{{t = t^{ * } }} = 0$. Note that, $t^{ * }$, is the time on the *x* axis at which the flexion point appears. Thus, using Eq. 1 together with the later condition we demonstrated that the kinetic of doxorubicin transverse movement, $k_{\text{m}}$

2 $$ k_{\text{m}} \cong \left( {\frac{{{\text{d}}\Updelta \tilde{I}}}{{{\text{d}}t}}} \right)_{{t = t^{ * } }} = cd\left( {1 - \frac{d}{b}} \right)\exp \left[ { - \frac{d}{b - d}\ln \left( { - \frac{{ab^{2} }}{{cd^{2} }}} \right)} \right] $$

### Hoechst 33258 diffusion across the cell membrane

Similar steps as done above were followed prior to adding 2 μl of Hoechst (*E*_x_/*E*_m_ = 355/460) at the required concentrations in the 200 μl wells containing PBS–doxorubicin and cells. The quantum yield of Hoechst changes dramatically upon interacting with cells and it was possible to measure its intensity over time using the fluorescent plate reader. Hoechst intensity displayed two trends, a strong exponential-like increase over short period of times (~5 min) followed by a linear trend afterward (Fig. 4c). A model was devised to take into consideration intensity changes based on a two compartments model. We assumed that the first increased was related to the amount of probes, $n_{\text{m}} (t)$, interacting with the membrane (compartment 1) [19], written as:

3 $$ {\text{d}}n_{\text{m}} /{\text{d}}t = k_{1} (N_{\text{m}} - n_{\text{m}} ) $$

In this context, $N_{\text{m}}$, is the total amount of Hoechst probes that can interact with the membrane and, $k_{1}$, the kinetic of interaction. We modelled the second trend as a linear accumulation of probes from the membrane into the cytosol (compartment 2), written as:

4 $$ {\text{d}}n_{\text{c}} /{\text{d}}t = k_{\text{m}} n_{\text{m}} $$

In this last context, $n_{\text{c}}$, is the total amount of Hoechst probes accumulating into the cytosol and, $k_{\text{m}}$, the kinetic of accumulation. As the quantum yield switched from near-zero to very-high value intensities we assumed a linear relationship between Hoechst intensity, $I(t)$, and Hoechst interacting with cells, $n_{\text{m}} + n_{\text{c}}$, such that:

5 $$ I(t)\sim n_{\text{m}} (t) + n_{\text{c}} (t) $$

Using Eqs. 3, 4, and 5 lead to:

6 $$ I(t)\sim N_{\text{m}} \left[ {k_{\text{m}} t + \left( {1 - \frac{{k_{\text{m}} }}{{k_{1} }}} \right)\left( {1 - {\text{e}}^{{ - k_{1} t}} } \right)} \right] $$

Using SigmaPlot software, experimental results were compared against Eq. 6 (Fig. 4c, *R* ^2 ^> 0.970 was the selection criteria) to deduce $k_{\text{m}}$ for Hoechst. Given the potential overlap between Hoechst emission and Dox absorption we carried out further analysis to determine whether FRET (fluorescence resonance energy transfer) was taking place. However, we did not measure any FRET levels in our experiments (data not shown).

### DiBac4(3) diffusion across the cell membrane

Similar steps as done above were followed prior incubating 2 μl of DiBac4(3) (*E*_x_/*E*_m_ = 485/590) (Sigma) required concentrations in the 200 μl wells containing PBS–doxorubicin and cells. The quantum yield of DiBac4(3) changes dramatically upon interacting with cells and it was possible to measure its intensity after a 40 min period using the fluorescent plate reader. Similarly done as above, no FRET was detected between DiBac4(3) and doxorubicin.

### Endocytosis measurement using FM210

Similar steps as done above were followed prior to incubating 2 μl of FM210 (*E*_x_/*E*_m_ = 485/620) (Sigma) required concentrations in the 200 μl wells containing PBS–doxorubicin and cells. The quantum yield of FM210 changes dramatically upon interacting with cells and it was possible to measure its intensity over time using the fluorescent plate reader. The slow increase in membrane endocytosis allows one to measure directly the kinetic of endocytosis with a one compartment model as described elsewhere [20, 21]. Similarly done as above, no FRET was detected between FM210 and doxorubicin.

### Statistical analysis

In graphs or plots error bars represent the standard deviation. The sign “*” denotes *p* value <0.05 between data. As Fig. 7a contains many histograms, the data signification is given literally directly within the legend. Number of replicate varied between three and ten depending on the experiment.

## Results

### Determination of doxorubicin sensitivity in K562 and DRK562 cell lines

**Fig. 1:**
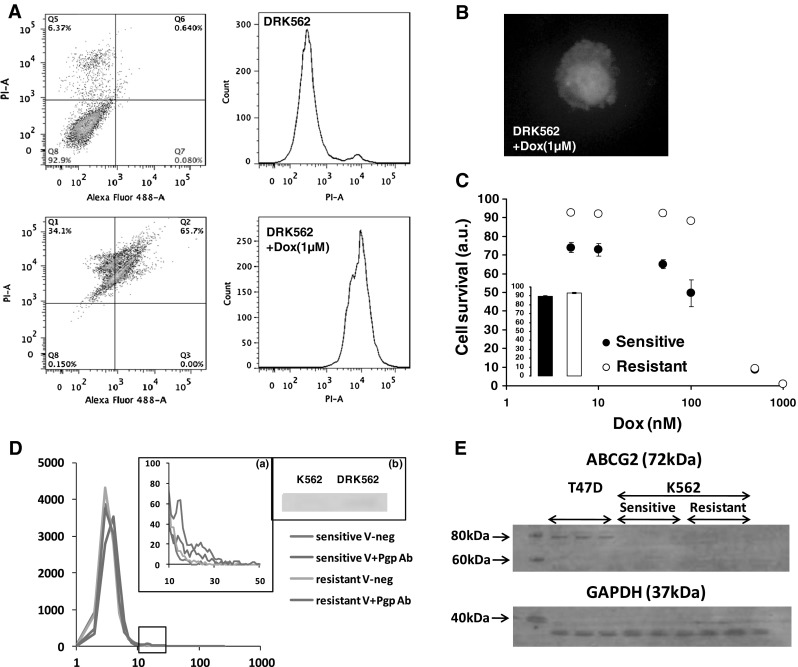
**A** Assessment of membrane permeability using propidium iodide (PI-A, *Y* axis) and phosphatidylserine exposure using annexin-V (Alex Fluor 488-A, *X* axis) of doxorubicin-treated (*bottom*) and non treated (*top*) drug-resistant K562 (DRK562) cells. Note the shift in fluorescence intensity between the doxorubicin-treated samples and control. **B** Confirmation of (**A**) using fluorescence microscopy. The *green* depicts annexin V in the outer leaflet of the cell membrane whereas the *orange–red* colour shows propidium iodide interacting with DNA. **C** Effect of doxorubicin concentration on cell survival for an overnight treatment. The *inset* represents the impact on survival of incubating cells in RPMI without FBS, no appreciable changes were noted meaning that it is doxorubicin that kills cells and not the experimental handling of cells. **D** FACS determination of Pgp expression levels in drug resistant DRK562 and drug sensitive K562 cells. *Inset* (*a*) provides a magnification of the region of interest selected by a square on the main figure. The legends containing the suffix “−neg” correspond to negative controls (i.e., no Pgp antibody incubated) whereas those containing the suffix “+Pgp-Ab” corresponds to samples where the Pgp antibody was incubated. In either case, we compared “sensitive” and “resistant” cell. *Inset* (*b*) shows the protein expression levels of Pgp. A very thin band can be distinguished for DRK562 confirming that some cells (but not all—see *inset* (*a*)) express Pgp. **E** Determination of ABCG2 expression levels in K562 and DRK562 compared to T47D cells

To compare the ability of drugs to cross the membrane in either resistant or sensitive cells we generated our own K562 drug resistant cell line as described in the protocol section. To address the level of resistance we measured the level of cell death in the presence of an increasing concentration of doxorubicin using two markers of apoptosis, firstly propidium iodide to probe membrane permeability and secondly, annexin V to probe phosphatidylserine outward translocation. The fluorescence intensity of propidium iodide and annexin VI were measured by FACS analysis (Fig. 1A). The results of the FACS showed populations of cells in four quadrants of scatter diagrams. Cells in quadrant 4 (Q4) were considered to be viable and these data were recorded for statistical analysis, but cells in other quadrants which showed increased staining with either annexin V or PI were either apoptotic or necrotic and therefore not considered viable. PI fluorescence histograms Fig. 1A also illustrate the presence of two distinct populations in the FACS results, as defined by the positive (lower right panel) and negative (upper panel) controls. Results were further confirmed by confocal microscopy (Fig. 1B). To determine the effective concentrations ($EC_{50}$), experimental results measuring cell death were matched against a logistic equation (see the fourth paragraph of “Materials and methods” section) (Fig. 1C) in either sensitive or resistant case. As expected we found K562 cells more sensitive to doxorubicin ($EC_{50} = 186 \pm 80\,{\text{nM}}$; $R^{2} = 0.956$) compared to homemade resistant DRK562 ($EC_{50} = 250 \pm 5\,{\text{nM}}$; $R^{2} = 0.999$). Note that a difference in Hill coefficients, *H*, was found between sensitive ($H = 2.1 \pm 1.2$) and resistant ($H = 3.2 \pm 0.1$) cells. In order to address Pgp expression following doxorubicin selection we FACS-analysed and compared K562 and DRK562. We found that only a very small fraction of resistant cells express Pgp (Fig. 1D (a, b)). This result was confirmed using western blot (Fig. 1D (b)). So the shift in effective concentrations is unlikely to results from the single expression of Pgp. In order to exclude another potent doxorubicin transporter we also looked at ABCG2 expression levels and found no expression in our cells compared to T47D cells [22] (Fig. 1E).

### Membrane adsorptions of doxorubicin on sensitive and resistant cells are similar at time zero but differ at longer time scale

**Fig. 2:**
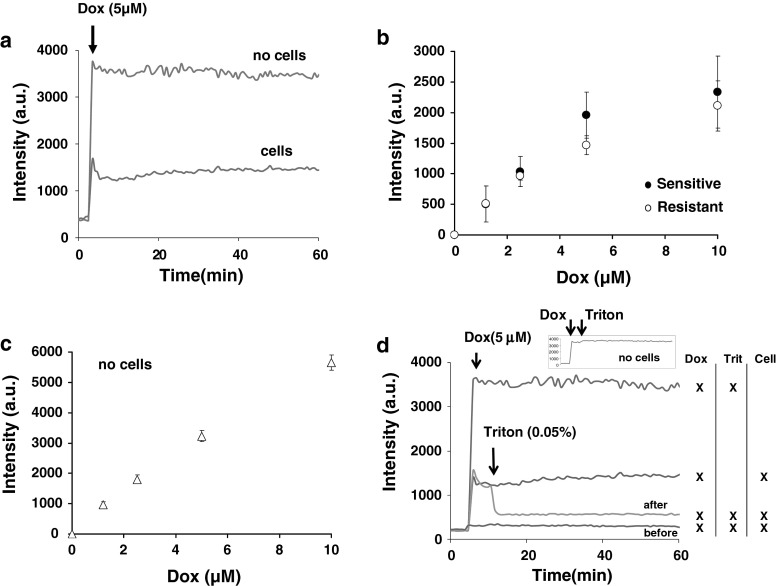
**a** Measure of fluorescence intensity changes upon doxorubicin incubation in the presence or absence of cells. **b** Determination of the intensity drop immediately after doxorubicin incubation in sensitive and resistant cells. **c** Linear relationship between the drops in fluorescence intensity and the concentration of doxorubicin used. **d** Effect of triton mediated alteration of membrane permeability and subsequent DNA binding on doxorubicin fluorescence in sensitive or resistant cells. The sigmoid-like increase in fluorescence intensity is not observed anymore in triton treated cells

To assess the interaction of doxorubicin with the membrane we measured the bleaching of doxorubicin fluorescence in the presence of cells (Fig. 2a). Based on the fluorescent signal changes we estimated the binding affinity of doxorubicin using a classical binding equation (Fig. 2b). Note that doxorubicin self-quenching in solution did not occur, or was negligible for the range of concentrations used (Fig. 2c). Thus, the changes in intensity were truly related to doxorubicin interacting with cells.

As doxorubicin is quenched upon DNA interaction in model vesicular systems, the drop in intensity in our cells could be due to this specific interaction. However the use of triton X-100 making the cell membrane more permeable allowed a further drop in intensity (Fig. 2d), suggesting that the slow changes in the intensity profile observed along the time (Fig. 2a) includes also the information regarding the interaction between doxorubicin with the membrane.

Finally, we matched the results from Fig. 2b against a classical-binding equation (similar to Eq. 8 see thereafter). Results did not show huge differences between sensitive and resistant cells: $K_{\text{sens}} = 6.4 \pm 1.3\,\mu {\text{M}}$ ($R^{2} = 0.980$) in drug sensitive cells versus $K_{\text{res}} = 7.0 \pm 0.5\,\mu {\text{M}}$ ($R^{2} = 0.994$) in drug resistant ones. Similarly the number of binding sites ($B$) deduced differed only by 10 % $B_{\text{sens}} /B_{\text{res}} \cong 1.10$ ($B_{\text{sens}} = 3979 \pm 749$ and $B_{\text{res}} = 3583 \pm 96$).

**Fig. 3:**
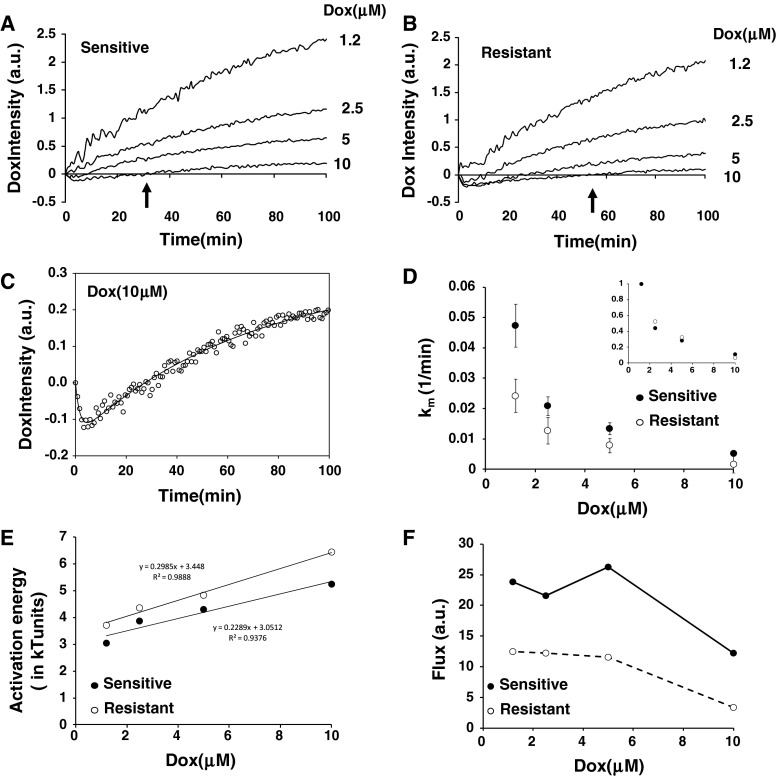
**a** Changes in doxorubicin intensity as a function of time and doxorubicin concentration in sensitive cells. **b** Changes in doxorubicin intensity as a function of time and doxorubicin concentration in resistant cells. **c** Data fitting using Eq. 2 (see Materials and methods). **d** Determination of the kinetic of doxorubicin transverses movement as a function of its concentration in drug sensitive or resistant cells. **e** Determination of activation energies assuming the transverses kinetic of doxorubicin in (**d**) follow an Arrhenius-like Law. **f** Estimation of doxorubicin influx into sensitive and resistant cells

Thus adsorptions of doxorubicin at time zero are similar in either cell types. However, shortly after the incubation data show that the drug will remain in the membrane for longer periods of time in drug resistant cells compared to sensitive ones (Fig. 3a, b)—see below.

### The kinetic of doxorubicin’s transverse movement across the membrane is inversely related to the amount of doxorubicin adsorbed in the membrane

After the initial abrupt changes in doxorubicin fluorescence we monitored the changes in fluorescence intensity as a function of time. We found that depending on doxorubicin concentration, the intensity was biphasic. An initial decrease followed by an increase in fluorescence (Fig. 3a, b). Moreover, there was a clear difference between drug sensitive and resistant cells. In particular, the fluorescence recovery upon binding was slower in drug resistant cells (arrows in Fig. 3a, b). We assumed that the biphasic changes over short period of times, i.e. fluorescence extinction and recovery within the first 30 min, were related to drug binding the membrane (extinction) followed by diffusion across the membrane to reach the cytoplasm (recovery). This period was followed by a slow exponential-like increase that was assumed to be related to drugs interacting with DNA (slow re-extinction).

It is notable from Fig. 3a and b that the kinetic changes in fluorescence intensities were inversely related to the extracellular concentration of doxorubicin. Thus, incorporating more doxorubicin into the cell membrane does not mean better and/or quicker intracellular delivery of the drug, but the contrary. As the amount of doxorubicin bound to membrane is close between drug resistance and sensitive cells (Fig. 2b) and that the fluorescence intensity of doxorubicin varies linearly with the regime of concentrations used (Fig. 2c), the changes observed were associated with the ability of doxorubicin to cross the membrane. It is also noteworthy that such ability to cross the membrane is delayed in doxorubicin resistant cells compared to sensitive cells (see arrows in Fig. 3a, b).

The slope of intensities of the fluorescence recovery were then determined and plotted against doxorubicin concentrations (Fig. 2d). We found overall that the kinetics of fluorescence recovery were remarkably slow $\sim 10^{ - 2} \min^{ - 1}$ in either cell type. This time scale is much longer to what was found previously in vesicular model systems [23]. Albeit the kinetic of fluorescence recovery was slower in drug resistant cells compared to sensitive ones, the direct effect of doxorubicin concentrations were similar in either cell types (inset, Fig. 3d). Using Arrhenius’ Law it was also possible to estimate a hypothetic energy barrier required to cross the membrane as a function of doxorubicin concentration (Fig. 3e). The possible role of a variation in the energy barrier upon doxorubicin suggested that membrane biophysical aspects may be taken into consideration. In this context, a difference of $\sim k_{\text{B}} T$ seems to make a difference between sensitive and resistant cells. Note that in “$k_{\text{B}} T$”, $k_{\text{B}}$ stands for the Boltzmann’s constant and $T$, for the absolute temperature.

Finally, as doxorubicin seems to affect its own entry, doxorubicin influx into cells was determined (Fig. 3f) by multiplying results from Fig. 2b (amount of doxorubicin bound to membrane initially) by those of Fig. 3d (kinetics of transverse movement). Figure 3f shows that doxorubicin influx is, as expected, smaller in drug resistant cells compared to drug sensitive ones and that both influx figures drop significantly when the external concentration of doxorubicin reaches 10 μM.

### Doxorubicin impacts negatively on the membrane adsorption of Hoechst

**Fig. 4:**
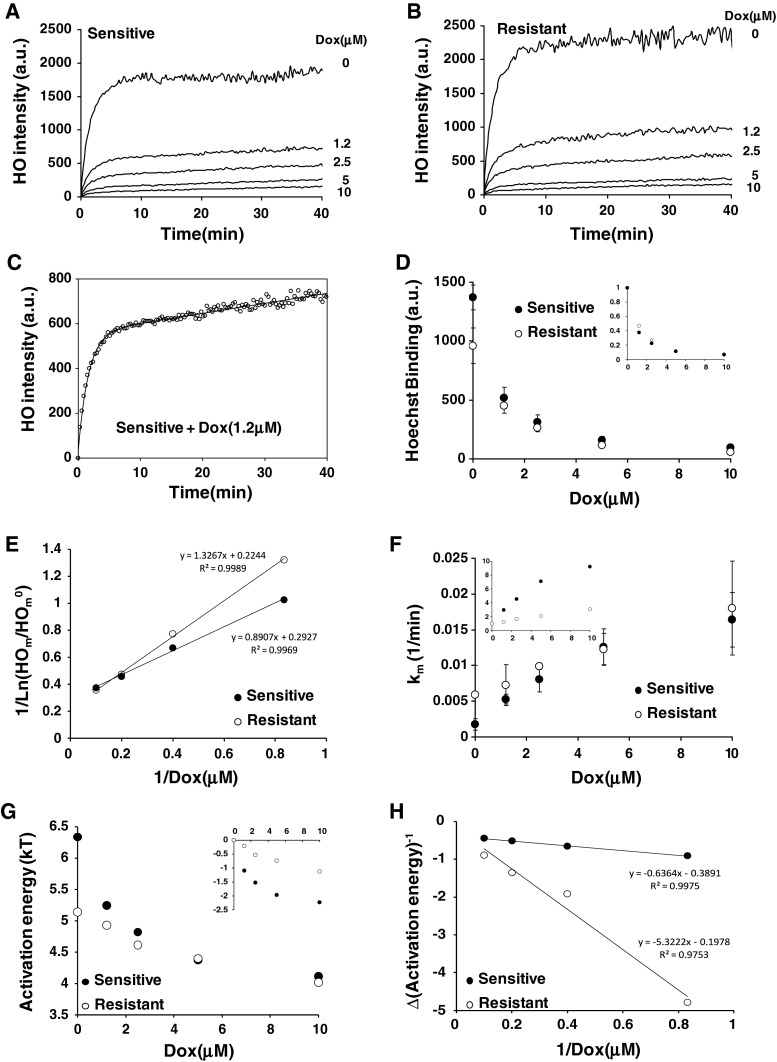
**a** Hoechst intensity as a function of time affected by a 15 min pre-treatment with doxorubicin in sensitive cells. **b** Hoechst intensity as a function of time affected by a 15 min pre-treatment with doxorubicin in resistant cells. **c** Data fitting using Eq. 6 (see Materials and methods). **d** Amount of Hoechst bound to cells immediately after injection as a function of doxorubicin pre-treatment for 15 min. **e** Lineweaver–Burk plot of data from (**d**). **f** Determination of the kinetic of Hoechst transverses movement as a function of doxorubicin pre-treatment in drug sensitive or resistant cells. **g** Determination of activation energies assuming the transverses kinetic of doxorubicin in (**f**) follow an Arrhenius-like Law. **h** Lineweaver–Burk plot of data from (**g**) assuming that membrane doxorubicin is responsible for the changes observed in the activation energy

Doxorubicin stays in membrane for long period of time. If such a long residency time in membrane is responsible for impeding doxorubicin influx, this should hold for any other molecules. We decided to use Hoechst as its quantum yield (i.e., fluorescence intensity) changes enormously when the probe interacts with hydrophobic partner. Such property facilitated modelling of Hoechst interacting with cells. Doxorubicin was pre-incubated for 15 min before Hoechst. In these conditions doxorubicin impeded Hoechst fluorescence in either sensitive or resistant cell lines (Fig. 4a, b) and Eq. 6 (see Materials and methods section) was matched against the experimental data (Fig. 4c). Albeit the amount of Hoechst initially bound to drug resistant cells was slightly higher (Fig. 4d), Hoechst binding to membrane decreased similarly but identically in the two cell lines as doxorubicin concentration increased (inset, Fig. 4d).

To determine whether pre-accumulation of doxorubicin in membrane resulted in a decrease in binding energy of Hoechst to the membrane, we assumed that the amount of Hoechst in membrane, ${\text{HO}}_{\text{m}}$, was a function of doxorubicin bound to the membrane, ${\text{Dox}}_{\text{m}}$, expressed by Arrhenius’ Law under the form:

7 $$ {\text{HO}}_{\text{m}} = {\text{HO}}_{\text{m}}^{0} \times {\text{e}}^{{b \times {\text{Dox}}_{\text{m}} /k_{\text{B}} T}} $$

Membrane bound doxorubicin was previously related to doxorubicin in solution, Dox, under the form:

8 $$ {\text{Dox}}_{\text{m}} = B \times {\text{Dox}}/(K + {\text{Dox}}) $$

Using the former and later equations it follows that:

9 $$ \ln ({\text{HO}}_{\text{m}} /{\text{HO}}_{\text{m}}^{0} ) = (b \times B)/k_{\text{B}} T \times {\text{Dox}}/(K + {\text{Dox}}) $$

By challenging the data from Fig. 4c against a Lineweaver–Burk plot, it was possible to determine whether doxorubicin binding coefficients in drug sensitive or resistant cells were similar to the ones determined previously from Fig. 2b. Only in the case of resistant cells were the binding coefficients very close, $K_{\text{res}} = 7.0\,\mu {\text{M}}$ in Fig. 2b versus $K_{\text{res}} = 6.5\,\mu {\text{M}}$in Fig. 4d or e. In sensitive cells, however, the binding coefficients were clearly different: $K_{\text{sens}} = 6.4\,\mu {\text{M}}$ in Fig. 2b versus $K_{\text{sens}} = 3.0\,\mu {\text{M}}$in Fig. 4d or e.

The similarity between the binding coefficients for resistant cells suggests that the impact on the membrane insertion of Hoechst is reflected by doxorubicin adsorption. Thus, the data support a linear causal link in this case. On the contrary, the lower binding coefficient in drug sensitive cells for Hoechst suggests that the membrane is made more sensitive to Hoechst by doxorubicin pre-incubation. As relatively less Hoechst can integrate into the membrane following doxorubicin pre-incubation in sensitive cells comparatively to drug resistant cells, this suggests that the membrane is, in a sense, made more “rigid” to Hoechst insertion. Thus, the results may suggest that the packing of lipid membrane (surface pressure) at least in the outer leaflet and induced by the pre-adsorption of doxorubicin in the membrane is likely to be central to the membrane adsorption of Hoechst.

In conclusion, pre-adsorption of doxorubicin seems to impact more strongly on Hoechst adsorption in sensitive cells than in resistant cells.

### Doxorubicin impacts positively on the transmembrane kinetic of Hoechst

Contrary to doxorubicin, the kinetic of Hoechst’s transverse movement increased as doxorubicin concentration increased (Fig. 4f). In this case, sensitive cells seemed to be more responsive than resistant ones (inset, Fig. 4f). An energy activation was then determined from Fig. 4f. It was found that the energy drops rapidly by value $\sim k_{\text{B}} T$ for micro-molar ranges of doxorubicin in drug sensitive cells (Fig. 4g). Ten times more doxorubicin was needed to observe a similar effect in drug resistant cells (inset, Fig. 4g). In order to determine how the effects from Fig. 4f were related to the amount of doxorubicin adsorbed in membrane (Eq. 8), we assumed that the drop in the activation energy was proportional to the amount of doxorubicin presents in the membrane. We carried out a similar analysis using the same formula as for the case of Hoechst above including a Lineweaver–Burk plot to determine $K_{\text{res}}$ and $K_{\text{sens}}$ (Fig. 4h). In this context, we found $K_{\text{sens}} = 1.7\,\mu {\text{M}}$ and $K_{\text{res}} = 27.9\,\mu {\text{M}}$. As a result Hoechst’s transverse movement is more impacted by doxorubicin pre-treatment in sensitive cells ($K_{\text{sens}} = 1. 7\,\mu {\text{M}}$) than in resistant cells ($K_{\text{res}} = 27.9\,\mu {\text{M}}$).

If one devises the membrane as a two compartment model namely outer and inner leaflets, the results suggest strongly that in drug resistant cells Hoechst transverse movement is impacted from the outer leaflet. On the contrary, both inner and outer leaflets seem important for drug sensitive cells.

To appreciate the differential contribution of either leaflets in drug sensitive and resistant cells endocytosis was measured.

### Fluid phase endocytosis is altered upon doxorubicin incubation

**Fig. 5:**
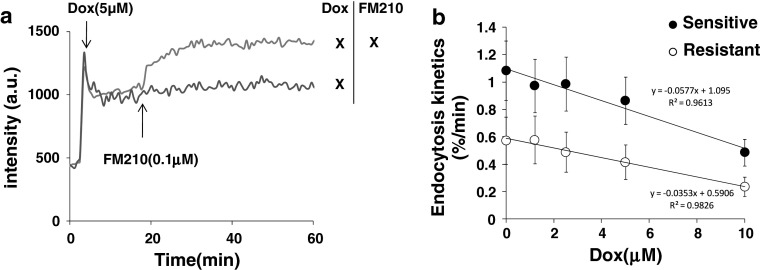
**a** Intensity changes associated with incubation of FM2-10 in the presence of doxorubicin. **b** Kinetics of fluid phase endocytosis as a function of doxorubicin pre-treatment

Previous works have demonstrated that fluid phase endocytosis is related to the differential surface tensions between membrane leaflets [21]. In particular, it was demonstrated (in K562 cells) that the kinetic of membrane endocytosis is proportional to the tension asymmetry [21]. Hence, measuring how endocytosis kinetics are altered upon treatments provide an idea of how membrane leaflets are affected. We carried out doxorubicin pre-incubation for 15 min and incubated FM210 in place of Hoechst (Fig. 5a).

It was found that doxorubicin has a strong effect in both drug sensitive and resistant cells (Fig. 5b). These results also confirmed that doxorubicin affects the membrane for long period of times. The near linear drop in the kinetics of endocytosis following doxorubicin incubation suggests that doxorubicin accumulation in the membrane reverses the endogenous difference in surface tensions. As the difference in surface tensions is the result of the lipid asymmetry in these cells (K562), that is around ~1–3 % [21, 24], the slope from the figure allows one to determine that the endogenous value of the lipid asymmetry decreases from its initial value by ~4–6 % per μM of doxorubicin incubated.

### DiBAC_4_(3) cellular accumulation is affected in drug sensitive and resistant cells following doxorubicin pre-treatment

**Fig. 6:**
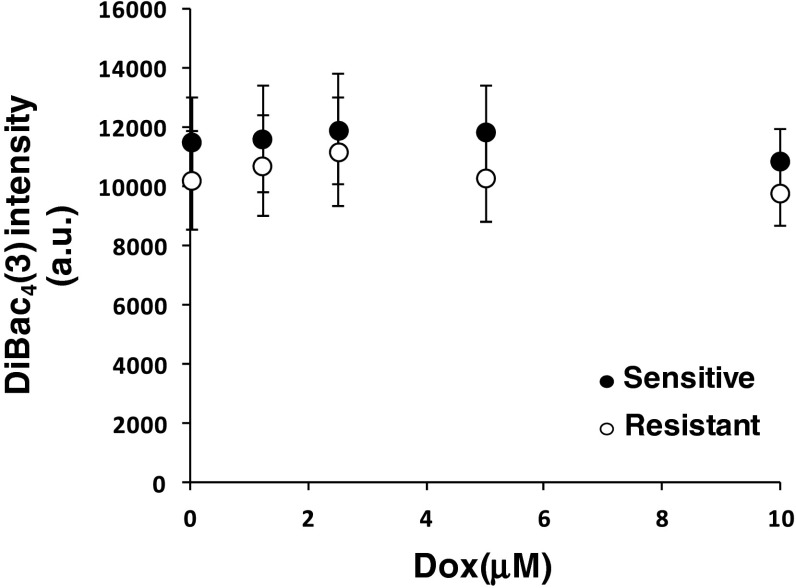
DiBac fluorescence intensity over a long exposure time as a function of doxorubicin pre-incubation for 15 min

As the result obtained could be related to changes in membrane potential mediated by doxorubicin pre-incubation, DiBAC_4_(3) was used to probe the electrical field across the membrane driven by the membrane potential of cells. DiBAC_4_(3) is well characterised and accumulate in cells with low membrane potential in an almost linear way [25]. In order to avoid miss reading due to doxorubicin pre-incubation and possible impact on the ability of DiBAC_4_(3) to traverse the membrane, we focused on DiBAC_4_(3) intracellular accumulation over long period of times, after 40 min incubation (Fig. 6). No significant differences were found between cell lines with or without doxorubicin pre-incubation, suggesting that the membrane potential is unaltered due to doxorubicin incubation.

### Statin and proton pump inhibitors sensitize drug resistant cells to doxorubicin

In resistant cells that do express drug transporters, chemicals can be used to re-sensitize cells to drugs. Some chemicals interact directly with transporters but it is not excluded that they could generate a dual effect interfering with the membrane properties as well. Among these chemicals statin-derived compounds commonly prescribed to inhibit the rate limiting enzyme (HMG-CoA) of the mevalonate pathway interact with Pgp [26] to resensitize cells to drugs [27].

**Fig. 7:**
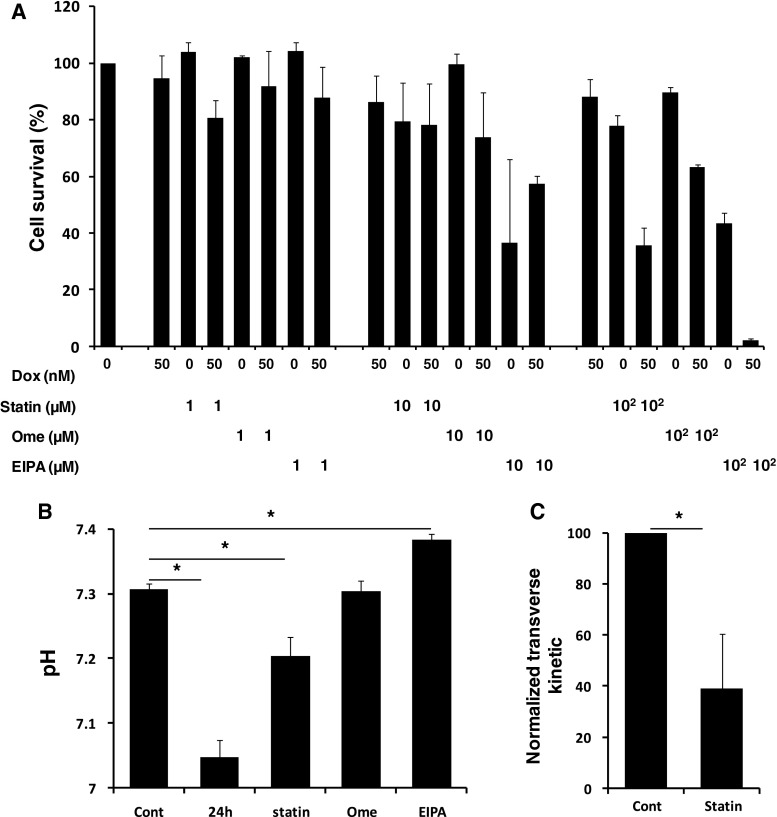
**a** Cell death induced by a 24 h incubation of atorvastatin, omeprazole or EIPA alone or in conjunction with a further 24 h treatment with doxorubicin (total incubation of 48 h). Results have been normalised by cells without treatment. Both the drug added and the dose of that drug are significant (*p* value < 0.05). The addition of atorvastatin, EIPA, or omeprazole and EIPA are always significantly different to the addition of no drug (*p* value < 0.05), and pre-treatment with either 10 or 100 μM of any drug was significantly different to the 1 μM dose (*p* value < 0.05). **b** Extracellular pH of DRK562 cell culture medium measured shortly after renewal (Cont) or after 24 h (24 h). The effect on the extracellular pH of atorvastatin (statin), omeprazole (ome) or EIPA are also given for comparison, all incubated at 100 μM after 24 h incubation. **c** Changes in the kinetic of Hoechst transverse movement in DRK562 after 24 h of atorvastatin (100 μM) incubation

We decided to investigate how atorvastatin could resensitize DRK562 to doxorubicin without involving drug transporters. In order to compare the effect of atorvastatin we used omeprazole and EIPA based on their ability to kill cells by blocking proton export [28–30] and to resensitize cells to drugs in drug transporter expressing [31] and non-expressing cells [32]. By pre-incubating DRK562 cells in the presence of these drugs for 24 h (step one), followed by a further 24 h incubation with 50 nM doxorubicin (step two). We found that 100 μM of atorvastatin is more efficient than omeprazole at killing DRK562 cells but less efficient than EIPA both employed at 100 μM (Fig. 7a). We verified that at the later concentration omeprazole and EIPA have an effect on proton export by measuring the extracellular pH after the initial 24 h incubation with DRK562 (Fig. 7b). To our surprise, we also measured a similar trend in the presence of atorvastatin but that seems to be less efficient than proton pump inhibitors (Fig. 7b). Finally using Hoechst it was apparent that the transverse movement of Hoescht was twice as long with atorvastatin suggesting a drop in membrane fluidity (Fig. 7c). It is noteworthy that the effect of atorvastatin on cell death is unlikely to be solely related to pH changes (Fig. 7b) as otherwise omeprazole would have been more effective in killing cells. Thus, membrane fluidity and related surface pressure seem key for mediating cell death in the presence or not of doxorubicin. Thus, when using drug resistance sensitizers it is imperative to study membrane properties as well as sensitizer interaction with Pgp.

## Discussion

The potential role of membrane composition and related biophysical properties has been suggested many times in drug resistance and is reviewed in [12]. Albeit the membrane has received much less attention than drug transporters over the years, the profound entanglement between the membrane biophysical properties and Pgp [8, 10, 11, 14, 33] calls for a clarification. In addition, there is now enough evidence demonstrating that the surface pressure of the membrane is an incidental “target” for some antineoplasic drugs [34–41] leading to the aggregation of apoptosis receptors to activate death pathways [36–38, 41–45]. This suggests that alteration of membrane fluidity may be paramount in drug resistant cells. Finally, the recent discovery that statin-derived drugs reverse drug resistance in Pgp-expressing cells [46] is a further element pointing the importance of the membrane in drug resistant cells.

To be able to work with cells and avoid the pitfall discussed in the introduction, we made our doxorubicin resistant cells so that Pgp and ABCG2, another doxorubicin transporter [22], were absent. Naturally these results do not rule out the possibility that another doxorubicin transporter that was not detected could be involved. However, this point is unlikely as: (i) the intracellular influx of doxorubicin does not increase—but decreases—as the external concentration of doxorubicin increases (Fig. 3f), which is contrary to what is expected from protein saturation of drug pumps; (ii) in our home-made drug resistant cells Hoechst is oblivious of the inner leaflet that should be central to mediate drug resistance following the Pgp scheme assuming a drug handling mechanism [6].

Herein, we measured how doxorubicin pre-incubation affects its own entry into cells and interactions between Hoechst and the membrane. Hoechst was chosen as it is often used to demonstrate Pgp-mediated drug resistance [47, 48].

We show here that with incubation of doxorubicin, transverse movement slows down dramatically when the concentration of the drug increase in either sensitive or resistant cells. The increase of the residence time of doxorubicin in the membrane affects Hoechst binding to the membrane. However, the drop in Hoechst binding does not affect Hoechst’s transverse movement as it crosses the membrane more rapidly. It seems that doxorubicin pre-incubation helps Hoechst to traverse the membrane. A very simple idea could be that a higher surface pressure from the outer leaflet due to doxorubicin pre-incubation could push Hoechst to traverse the membrane. The pushing mechanism can be imaged as an olive stone (the drug) pressed between two fingers (the lipids from the outer leaflet) and pushed away (across the membrane).

This model seems to stand out. Indeed, it is unlikely that an increase in global membrane fluidity induced by doxorubicin pre-incubation explain the “lubrication” observation, as an increase in fluidity should have facilitated Hoechst binding. Furthermore, this ability of doxorubicin to facilitate Hoechst entry does not seem to be correlated to the membrane potential of cells and related electromotive forces [49], as drug sensitive cells that are the most affected by doxorubicin do not show any appreciable changes in their membrane potential further to doxorubicin pre-incubation. The one biophysical parameter that is then left is the differential packing of lipids across the membrane. Accordingly, we also found that endocytosis is affected by doxorubicin in drug sensitive and resistant cells.

In this context and from results concerning Hoechst binding and transverse movement it is possible to estimate the resting membrane tension prior to incubating doxorubicin. In these conditions, in the expression of ${\text{HO}}_{\text{m}}$ (Eq. 7) the parameter $b$ would be expressed as: $b = - \sigma \times a_{\text{Dox}}$; where $\sigma$ is the tension and $a_{\text{Dox}}$ the cross-section of doxorubicin. As for drugs small enough the molecular weight (*M*_*W*_) is proportional to the molecular volume it follows: $a_{\text{Dox}} \sim \pi (3M_{{W_{\text{Dox}} }} /4\pi )^{2/3}$. Knowing the *M*_W_ of doxorubicin, it is thus possible to determine: $\sigma_{\text{sens}} \sim 5 \times 10^{ - 3} \,{\text{mN}}/{\text{m}}$ and $\sigma_{\text{res}} \sim 7 \times 10^{ - 3} \,{\text{mN}}/{\text{m}}$. The later values are similar to what is found ($\sim 3 \times 10^{ - 3} \,{\text{mN/m}}$) when cells have large reservoir of membrane [50, 51] and a large reservoir of membrane is expected for these cells [17, 21]. Accordingly the surface tension of the outer leaflet of sensitive cells would be lower than in drug resistant cells. This could explain the drop in Hoechst binding initially prior to incubating doxorubicin. Note that the values found are higher than the thermal value of the surface tension needed to impede the transverse movement of doxorubicin ($k_{\text{B}} T/a_{\text{Dox}} \sim 2 \times 10^{ - 4} \,{\text{mN}}/{\text{m}}$) and thus the results above are therefore coherent with a potential physical effect. Moreover, our energy values related to drug–membrane interaction are much higher than those linked to electromotive forces previously involved in drug resistant cells, see “Discussion” in [8]. Hence, the underlying physical mechanism must be central to drug delivery.

One point needs to be resolved, however. Let us suppose that the outer leaflet is altered between drug sensitive and resistant cells as suggested. If that is the case why only a marginal effect is observed on doxorubicin adsorption between cell lines (Fig. 2b)? The answer comes from a comparison between Figs. 3d and 4f. In these, the transverse movement kinetic in control conditions is ten times longer for doxorubicin than Hoechst. This seems to suggest that doxorubicin has stronger affinity with the cell membrane compared to Hoechst. Therefore, doxorubicin adsorption would be dictated chiefly by its hydrophobic character hence the lack of drastic differences between binding coefficients in sensitive and resistant cells (Fig. 2b).

Finally one can interpret the results concerning the kinetics of endocytosis as a consequence of changes in the differential packing of lipids across the membrane. It was demonstrated in K562 drug sensitive cells that the difference in surface tensions—i.e., the differential packing of lipids across the membrane—is around $\Updelta \sigma_{\text{sens}} \sim 9 \times 10^{ - 4} \,{\text{mN}}/{\text{m}}$ [21]. As the kinetic of membrane endocytosis measured is proportional to the difference in surface tensions [17], this would mean that the same endogenous difference in surface tensions in drug resistant cells should be $\Updelta \sigma_{\text{res}} \sim 5 \times 10^{ - 4} \,{\text{mN}}/{\text{m}}$. The later value is relatively close to the thermal value of the surface tension needed to impede doxorubicin that in turn could explain why Hoechst is more or less oblivious of the inner leaflet in these resistant cell (recall that $K_{\text{res}} \sim 27.9\,\mu {\text{M}}$). Albeit a similar conclusion could be applied in theory to drug sensitive cells—namely that the inner leaflet should have a marginal impact on the drug transverse movement since $\Updelta \sigma_{\text{sens}} < \sigma_{\text{sens}}$—this is not confirmed experimentally (recall that $K_{\text{sens}} \sim 1.7\,\mu {\text{M}}$). Therefore, mechanisms other than those involving the membrane mechanical properties are very likely involved in this case. This fixes a definitive limit to the “olive stone” model that seems to be only coherent in drug resistant cells and underline the appropriateness of using living cellular systems to study drug resistance.

It is also important to underline that our system does not express Pgp and therefore our results cannot be fully transferred to Pgp-expressing cells. Furthermore, it is important to emphasize that we did not find an increase in endocytosis contrary to what was measured in Pgp-expressing cells [12].

To conclude we suggest here that the ability of chemicals to cross the membrane rely on the energy difference between outer and inner leaflets and that incubation of drug chemicals would affect this difference resulting in nonlinear complications. Finally, a clearer understanding of the physical biology of MDR is necessary to improve and initiate new therapeutic strategies.
